# PANoptosis in neurological disorders: from inflammatory cell death mechanisms to neuroprotective strategies

**DOI:** 10.3389/fnins.2026.1890903

**Published:** 2026-06-30

**Authors:** Hongbo Zhang, Ruoxin Tu, Dawei Zhang, Xiangyue Meng, Mengdi Yu, Shuo Ding, Chenhui Ma, Xinyue Qiu, Xiaoqi Yu, Hongna Yin

**Affiliations:** 1 Heilongjiang University of Chinese Medicine, Harbin, China; 2 Heilongjiang Academy of Traditional Chinese Medicine, Harbin, China; 3 The Second Affiliated Hospital of Heilongjiang University of Chinese Medicine, Harbin, China

**Keywords:** apoptosis, necroptosis, neurodegeneration, neurological diseases, PANoptosis, pyroptosis

## Abstract

PANoptosis is now regarded as an inflammatory form of programmed cell death (PCD). It reflects the coordinated involvement of apoptosis, pyroptosis, and necroptosis, usually through the PANoptosome in a shared pathological environment. This concept may be especially useful in neurological diseases. It helps explain why neuronal death, sustained inflammatory activation, and tissue injury often develop together and reinforce one another. Neural tissue is particularly sensitive to oxidative stress, mitochondrial dysfunction, immune-mediated inflammation, and blood-brain barrier disruption. These pathological changes are common in many forms of neural injury. Therefore, abnormal PANoptosis activation may provide a common mechanism linking different types of nervous system damage. This review summarizes the historical evolution, molecular mechanisms, disease-related roles, and intervention strategies of PANoptosis in neurological disorders. It focuses on PANoptosome assembly and key mechanistic nodes, including NOD-like receptor family pyrin domain-containing 3 (NLRP3), caspase-8, the receptor-interacting serine/threonine protein kinase 1 (RIPK1)/receptor-interacting serine/threonine protein kinase 3 (RIPK3)/mixed lineage kinase domain-like protein (MLKL) axis, gasdermin D (GSDMD), and Ninjurin 1 (NINJ1). It also highlights current translational limitations, such as disease heterogeneity, incomplete cell-specific validation, and insufficient clinical evidence.

## 1 Introduction

Neurological diseases include disorders that affect the brain, spinal cord and peripheral nerves. They include chronic degenerative diseases such as Alzheimer's disease (AD), Parkinson's disease (PD), Huntington's disease (HD) and amyotrophic lateral sclerosis (ALS), as well as acute injury-related diseases such as ischemic stroke (IS), subarachnoid hemorrhage (SAH), intracerebral hemorrhage (ICH) and spinal cord injury (SCI). A recent global burden study showed that disorders affecting the nervous system are now a major source of health loss worldwide. This means that neurological diseases are not only problems of one organ, but long-term conditions that can affect movement, cognition, speech, emotion and daily independence (GBD 2021 Nervous System Disorders Collaborators, 2024). Although neurological diseases arise from diverse etiologies, they often exhibit similar manifestations during their pathological progression. Specifically, neuronal death and inflammatory responses collectively contribute to the development of both acute central nervous system injuries and chronic neurodegenerative disorders. Furthermore, the structural components of the neurovascular unit—including neurons, glial cells, endothelial cells, and pericytes are susceptible to factors such as ischemia and hypoxia, protein misfolding, oxidative stress, inflammatory stimuli, hemorrhagic byproducts, or mechanical trauma (Kugler et al., 2021; Shi et al., 2021). Past research has often categorized cell death into relatively independent regulatory types, among which apoptosis, pyroptosis, and necroptosis are the three most frequently discussed forms of programmed cell death (PCD) in studies of neuroinflammation and neural injury (Galluzzi et al., 2018). Apoptosis is typically characterized by cell shrinkage, chromatin condensation, DNA fragmentation, and the formation of apoptotic bodies. The cell membrane remains relatively intact for an extended period, and thus, it generally does not result in the immediate release of massive cellular contents as seen in lytic cell death (Kerr et al., 1972). Pyroptosis is more inherently inflammatory. Its typical process involves the activation of the inflammasome, the cleavage of gasdermin D (GSDMD) by inflammatory caspases, the formation of pores in the cell membrane, the release of interleukin-1β (IL-1β), and osmotic cell lysis (Fink and Cookson, 2006; He et al., 2015). Necroptosis is typically mediated by molecules such as receptor-interacting serine/threonine protein kinase 1 (RIPK1), receptor-interacting serine/threonine protein kinase 3 (RIPK3), and mixed lineage kinase domain-like protein (MLKL). After being phosphorylated by RIPK3, MLKL is activated and contributes to plasma membrane damage. This membrane damage allows intracellular materials to escape from the cell. The released contents can then promote an inflammatory response (Cho et al., 2009; Wang et al., 2014). Recent evidence suggests that these three pathways should not be understood as fully separate processes. Their boundaries can become blurred in specific biological contexts. Caspase-8 illustrates this connection well, as findings from genetic mouse models and intestinal epithelial cells indicate that it can participate in the regulation of apoptosis, necroptosis, and pyroptosis. Thus, caspase-8 may function as an important regulatory link among different forms of programmed cell death (Fritsch et al., 2019). These findings have led researchers to reconsider cell death as a connected process. Rather than examining each pathway in isolation, current studies increasingly ask how different death pathways communicate with and influence one another.

The concept of PANoptosis emerged from this background. It describes a situation in which these pathways are activated in the same disease setting and are organized by multi-protein complexes (Christgen et al., 2020). This multi-protein complex is known as the PANoptosome. It integrates molecules such as NOD-like receptor family pyrin domain-containing 3 (NLRP3), apoptosis-associated speck-like protein containing a caspase activation and recruitment domain (CARD), hereafter referred to as apoptosis-associated speck-like protein containing a CARD (ASC), caspase-1, caspase-8, RIPK1, RIPK3, and MLKL, thereby enabling the mutual amplification of cell death and inflammatory responses (Christgen et al., 2020). In the nervous system, neurons have limited regenerative capacity, and once they die, they are difficult to fully replace. Microglia and astrocytes help clear damage and support tissue homeostasis. However, prolonged activation can change their function. In this state, they may continue to release inflammatory mediators, including IL-1β, IL-18, and tumor necrosis factor alpha (TNF-α). These mediators can further aggravate neural cell injury (Pandeya and Kanneganti, 2024). In neurological diseases, cell death is rarely just a final outcome. It can also connect inflammatory activity, immune changes, and tissue damage (Shi et al., 2021).

PANoptosis may help explain neurological diseases from a broader angle. It connects different forms of cell death with persistent inflammation and progressive tissue injury. This narrative review brings together evidence from mechanistic studies, disease models, and early therapeutic research to discuss PANoptosis in neurological disorders. The review begins with the development of PANoptosis as a concept, which arose from earlier studies of apoptosis, pyroptosis, and necroptosis as separate death pathways. It then explains how PANoptosome-centered complexes provide a framework for understanding inflammatory cell death. The main molecular events are also summarized. These include danger-signal sensing, PANoptosome assembly, caspase activation, GSDMD-mediated pore formation, RIPK1/RIPK3/MLKL signaling, and NINJ1-driven membrane rupture. In addition, we discuss the involvement of PANoptosis in major neurodegenerative diseases and acute neurological injuries. The review ends by considering emerging therapeutic strategies and the remaining difficulties in mechanistic validation and clinical translation.

## 2 PANoptosis from a historical perspective

The idea of PANoptosis developed from earlier work on three forms of PCD: apoptosis, pyroptosis, and necroptosis. Apoptosis was first described in 1972 by Kerr, Wyllie, and Currie based on histomorphological observations. They reported several characteristic changes, such as cell shrinkage, chromatin condensation, and the removal of cellular fragments by neighboring cells. This work was important because it showed that cell death could be an organized biological process, rather than a simple result of cellular collapse (Kerr et al., 1972). Apoptosis research then began to move from morphology toward molecular regulation. One important step came from the work of Vaux and colleagues. Using hematopoietic cells, they found that the B-cell lymphoma 2 (Bcl-2) gene helped cells survive and could cooperate with the MYC oncogene to immortalize pre-B cells. This finding supported the idea that cell death is not simply the result of external damage, but can be controlled by internal genetic programs (Vaux et al., 1988). Zou and colleagues later studied this process *in vitro*. They found that apoptotic protease activating factor 1 could activate caspase-3, but this required cytochrome c and deoxyadenosine triphosphate. These results provided important evidence for the molecular mechanism of the mitochondrial apoptotic pathway (Zou et al., 1997). Jänicke et al. (1998) reached a similar conclusion using caspase-3-deficient cells. After treatment with tumor necrosis factor or staurosporine, these cells showed changes in DNA fragmentation and classical apoptotic morphology. The study therefore strengthened the idea that apoptosis is not a random process, but depends on defined execution molecules (Jänicke et al., 1998). In 2018, the Nomenclature Committee on Cell Death updated the classification of regulated cell death. In this framework, apoptosis was placed under the category of non-lytic regulated cell death (Galluzzi et al., 2018). This established a unified terminological foundation for subsequent comparisons between apoptosis, pyroptosis, and necroptosis.

The study of pyroptosis developed along a different route from apoptosis. It first attracted attention in the context of infection and inflammatory responses. Brennan and Cookson found that macrophage death after Salmonella infection depended on caspase-1, but its morphology was different from classical apoptosis and showed inflammatory necrosis-like changes with the release of intracellular contents (Brennan and Cookson, 2000). In 2001, Cookson and Brennan formally proposed the term pyroptosis to describe this pro-inflammatory form of PCD (Cookson and Brennan, 2001). The understanding of pyroptosis was later connected with inflammasome research. In 2002, Martinon, Burns and Tschopp proposed the concept of the inflammasome. Their biochemical experiments showed that NOD-like receptor (NLR) related complexes could act as platforms for caspase-1 activation and promote the maturation of interleukin-1β (Martinon et al., 2002). Fink and colleagues further demonstrated in Salmonella-infected macrophages that caspase-1-dependent pyroptosis causes pore formation in the cell membrane, leading to osmotic imbalance and cell lysis. This connected inflammasome activation with lytic inflammatory cell death (Fink and Cookson, 2006). After 2013, pyroptosis research entered the stage of non-canonical inflammasome signaling. Hagar and colleagues found in mice that cytosolic lipopolysaccharide (LPS) could activate caspase-11 and cause endotoxic shock independently of membrane Toll-like receptor 4 (TLR4) (Hagar et al., 2013). Kayagaki and colleagues further showed that caspase-11 cleaves GSDMD, and that GSDMD is an important executor for pyroptosis and IL-1β maturation in non-canonical inflammasome signaling (Kayagaki et al., 2015). Around 2015, the discovery of GSDMD moved pyroptosis research from a caspase-1-dependent death model to a gasdermin-mediated pore-forming model. Shi and colleagues used genome-wide CRISPR-Cas9 screening in mouse bone marrow-derived macrophages and found that GSDMD deficiency made cells resistant to cytosolic LPS and canonical inflammasome ligand-induced pyroptosis, while also reducing IL-1β release (Shi et al., 2015). He and colleagues used quantitative mass spectrometry and gene knockout experiments and found that GSDMD participates in nigericin-induced NLRP3 inflammasome responses and is required for pyroptosis and IL-1β secretion (He et al., 2015). The proposal of necroptosis changed the traditional understanding of necrosis. In 2005, Degterev and colleagues identified a small molecule inhibitor of necroptosis named necrostatin-1 (Nec-1) through cell screening and mouse ischemic brain injury experiments (Degterev et al., 2005). This study showed that some necrosis-like cell death can be regulated by molecular drugs and may participate in delayed ischemic brain injury. In 2008, Degterev and colleagues further proved that the main target of Nec-1 is RIPK1, moving necroptosis from a morphological idea toward a defined signaling pathway (Degterev et al., 2008). Cho and colleagues reported in 2009 that RIPK1 interacts with RIPK3 to form a phosphorylation-dependent complex. This complex is involved in programmed necrosis and virus-related inflammation. Since then, the RIPK1-RIPK3 axis has become an important focus in necroptosis research (Cho et al., 2009). Wang and colleagues later placed MLKL downstream of RIPK3 in the necroptosis pathway. After phosphorylation by RIPK3, MLKL becomes active and injures the cell membrane. This membrane damage then results in necrotic cell death (Wang et al., 2014).

As research into these three pathways has deepened, researchers have gradually discovered significant intersections between them. In an influenza A virus (IAV) infection model, Nogusa et al. found that RIPK3 can induce not only necroptosis through MLKL, but also apoptosis mediated by Fas-associated death domain protein (FADD). This indicates that a single upstream stimulus can concurrently mobilize distinct cell death programs (Nogusa et al., 2016). Sarhan and colleagues also found under Yersinia infection and transforming growth factor-β-activated kinase 1 (TAK1) inhibition that caspase-8 is not only involved in apoptosis, but can also cleave GSDMD and gasdermin E (GSDME), thereby inducing pyroptosis-like cell death (Sarhan et al., 2018). Fritsch and colleagues found in genetic mouse models that enzymatically inactive caspase-8 caused embryonic lethality together with necroptosis and pyroptosis activation. Blocking MLKL together with ASC or caspase-1 rescued this lethal phenotype, indicating that caspase-8 is an important molecular switch among apoptosis, necroptosis and pyroptosis (Fritsch et al., 2019). On the basis of these studies, the concept of PANoptosis gradually took shape. In 2019, Malireddi and colleagues discussed studies on Z-DNA-binding protein 1 (ZBP1) and TAK1 and proposed that pathogen stimulation or inflammatory signaling can simultaneously engage pyroptosis, apoptosis and necroptosis through complexes involving RIPK1, RIPK3, FADD and caspase-8. They referred to this integrated process as PANoptosis (Malireddi et al., 2019). The significance of this naming is that these three death pathways are no longer viewed as completely independent parallel routes, but as pathways that can be coordinated by a shared platform under the same pathological stimulus. In 2020, Christgen and colleagues further proposed and validated the concept of the PANoptosome. In macrophages infected with IAV, vesicular stomatitis virus (VSV), *Listeria monocytogenes* or *Salmonella enterica* serovar Typhimurium, they detected the simultaneous activation of markers of pyroptosis, apoptosis and necroptosis. Protein interaction evidence also supported the formation of a multi-protein complexes (Christgen et al., 2020). In the same year, Malireddi and colleagues found in Yersinia infection studies that RIPK1 regulates the assembly of a RIPK1-PANoptosome. Loss of RIPK1 weakened PANoptosis-related protein activation and inflammatory responses, suggesting that PANoptosomes may be organized differently depending on the stimulus (Malireddi et al., 2020). After 2021, PANoptosome research expanded from a few infection models to multiple innate immune sensors. Lee and colleagues found in herpes simplex virus 1 (HSV-1) and *Francisella novicida* infection models that absent in melanoma 2 (AIM2) can form a complex with pyrin and ZBP1. This complex also contains ASC, caspase-1, caspase-8, RIPK1, RIPK3 and FADD, and can drive PANoptosis and host defense (Lee et al., 2021). This study showed that PANoptosis is not downstream of only one receptor, but can be organized by different innate immune sensors. In 2023, Sundaram and colleagues found that NOD-like receptor family pyrin domain-containing 12 (NLRP12) can form an NLRP12-PANoptosome in response to heme plus pathogen-associated molecular patterns (PAMPs) or TNF. This complex induced inflammasome activation, cell death and tissue pathology (Sundaram et al., 2023). This finding expanded PANoptosis from purely infectious stimuli to damage-related triggers such as heme, which is important for understanding cell death in hemorrhage, hemolysis and sterile inflammation. In 2024, Sundaram and colleagues further found that NOD-like receptor family CARD domain-containing 5 (NLRC5) can sense nicotinamide adenine dinucleotide (NAD^+^) depletion and form an NLRC5-PANoptosome to drive inflammatory cell death (Sundaram et al., 2024). In 2025, Sharma and colleagues found that NLRP3 is not only a canonical inflammasome sensor, but can also drive PANoptosome formation and PANoptosis under specific conditions (Sharma et al., 2025). These findings further expanded the sensor family of PANoptosomes.

**Figure 1:**
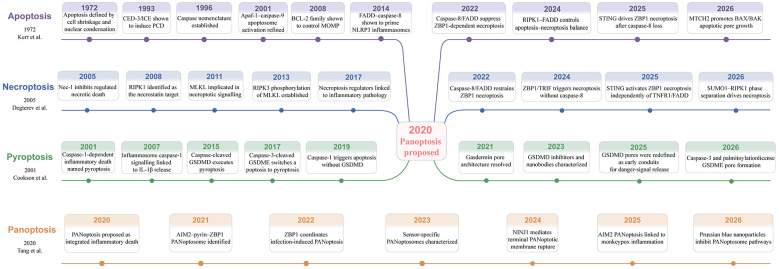
Historical milestones in programmed inflammatory cell death and the emergence of PANoptosis. This timeline summarizes major conceptual and mechanistic advances in apoptosis, necroptosis, pyroptosis, and PANoptosis. Early studies established apoptosis as a genetically regulated, morphologically distinct form of programmed cell death, whereas subsequent discoveries identified necroptosis and pyroptosis as regulated lytic death pathways driven by RIPK1/RIPK3/MLKL signaling and inflammasome–gasdermin activation, respectively. The recognition of extensive molecular crosstalk among caspases, inflammasomes, RIP kinases, gasdermins, and membrane-rupture machinery gradually challenged the traditional view that these pathways operate independently. In 2020, PANoptosis was proposed as an integrated inflammatory cell death program coordinated by PANoptosome complexes, providing a unified framework to explain the simultaneous activation of pyroptotic, apoptotic, and necroptotic machinery under infection, sterile inflammation, and tissue injury. Subsequent studies further expanded this field by identifying sensor-specific PANoptosomes, ZBP1- and AIM2-related regulatory nodes, NINJ1-mediated terminal membrane rupture, and disease-associated PANoptotic mechanisms, thereby redefining inflammatory cell death as a highly coordinated and context-dependent signaling network.

This historical process shows that the formation of PANoptosis went through three key stages. In the first stage, apoptosis, pyroptosis and necroptosis were discovered separately and explained at the molecular level. Researchers gradually realized that cell death differs not only in morphology, but also in execution mechanisms. In the second stage, molecules such as caspase-8, RIPK1, RIPK3, MLKL, GSDMD and inflammasomes were found to be connected across different death pathways. This made a single pathway explanation insufficient for complex inflammatory diseases. In the third stage, the concept of the PANoptosome was proposed and different sensor-based complexes were identified, making PANoptosis an integrated framework that connects pyroptosis, apoptosis and necroptosis (Figure 1).

## 3 Mechanism of PANoptosis

PANoptosis can be understood as an integrated inflammatory form of PCD driven by innate immune signaling. It is not a mechanical mixture of pyroptosis, apoptosis and necroptosis, but a coordinated process in which these pathways are activated through a shared death platform (Christgen et al., 2020). This platform is often called the PANoptosome. It works like a molecular convergence point that brings sensor proteins, adaptor proteins, caspases and necroptosis-related kinases together, so that cell death no longer proceeds through only one isolated route (Wang et al., 2022). To determine whether a cell undergoes PANoptosis, one should not rely on a single marker. Pyroptotic markers, apoptotic markers and necroptotic markers should be evaluated together, including GSDMD cleavage, caspase-3 or caspase-7 activation, and changes in RIPK3 and MLKL (Wang et al., 2022).

The first step of PANoptosis is usually the recognition of PAMPs or damage-associated molecular patterns (DAMPs). These signals may come from viruses, bacteria or fungi, and they may also come from nucleic acids, heme, cytokines or metabolic stress released after tissue injury (Sundaram et al., 2023). In influenza virus infection models, ZBP1 has been shown to recognize viral components and activate the RIPK1, RIPK3 and caspase-8 axis, thereby promoting inflammasome activation, apoptosis and necroptosis at the same time (Kuriakose et al., 2016). Later work further showed that retinoic acid-inducible gene I and mitochondrial antiviral signaling protein-mediated interferon-β signaling increases ZBP1 expression and enables ZBP1 to recognize viral ribonucleoprotein complexes (Kesavardhana et al., 2017). This means that ZBP1 does not work as an isolated sensor. It often requires upstream interferon signaling to place the cell in an alert state, after which PANoptosis-related complexes can be assembled more easily. In fungal infection models, ZBP1 was also shown to act as an upstream sensor that contributes to the combined activation of pyroptosis, apoptosis and necroptosis, suggesting that ZBP1-related PANoptosis is not restricted to viral infection (Banoth et al., 2020).

The PANoptosome does not have a fixed composition. Rather, it represents a flexible cell-death platform that can be reorganized according to the type of stimulus. Early studies on the PANoptosome showed that macrophages exposed to influenza virus, VSV, *Listeria monocytogenes* and *Salmonella enterica* serovar Typhimurium displayed markers of pyroptosis, apoptosis and necroptosis at the same time. These markers did not appear as separate events, but were associated with the formation of a multiprotein death complex (Christgen et al., 2020). Therefore, instead of viewing the PANoptosome as an upgraded version of one single death pathway, it is more accurate to regard it as a signal integrator formed under strong inflammatory stress. Within this integrator, ASC, caspase-8 and RIPK3 are often considered core structural anchors. Expansion microscopy and immunofluorescence studies showed that these molecules can colocalize at the single-cell level, which provides useful evidence for the formation of PANoptosome-like complexes (Wang et al., 2022). In different disease or injury contexts, the upstream sensors of the PANoptosome may change. For example, in HSV-1 and *Francisella novicida* infection models, AIM2 does not function only as a classical DNA inflammasome sensor. It also regulates pyrin and ZBP1, and forms an AIM2-PANoptosome together with ASC, caspase-1, caspase-8, RIPK1, RIPK3 and FADD. This complex promotes inflammatory cell death related to host defense (Lee et al., 2021). This finding suggests that PANoptosome assembly is not simply directed by one inflammasome sensor alone. Instead, it is more like a cooperative process among several sensors. In other words, when pathogen-derived or injury-derived signals are sufficiently complex, the cell may not choose only one pathway such as AIM2, NLRP3 or ZBP1. It may recruit several sensors and death-execution molecules into the same complex to eliminate infected or damaged cells more efficiently. In addition to infection, heme-related injury can also induce a specific type of PANoptosome. NLRP12 has been reported to sense the combined stimulation of heme and PAMPs, and to form an NLRP12-PANoptosome with molecules such as ASC, caspase-8 and RIPK3. In cell experiments and mouse disease models, this pathway was associated with increased inflammatory cytokine release, tissue injury and pathological damage (Sundaram et al., 2023). These findings are particularly informative for neurological diseases, because red blood cell lysis, free heme release, mitochondrial damage and sterile inflammation may occur in SAH, ICH and ischemia-reperfusion injury. Recent studies have further expanded the range of PANoptosome sensors. NLRC5 was found to sense NAD^+^ depletion-related stress, and to interact with NLRP12 and other PANoptosome components, thereby forming an NLRC5-PANoptosome and inducing PANoptosis (Sundaram et al., 2024).

After the PANoptosome is formed, cell-death signaling does not proceed in only one direction. Instead, it creates cross-activation among pyroptosis, apoptosis and necroptosis. The pyroptotic branch is particularly important in PANoptosis because it directly involves membrane pore formation and inflammatory cytokine release. A classical study using genome-wide screening, GSDMD knockout and rescue experiments showed that GSDMD is a common substrate of inflammatory caspases. Caspase-1, caspase-4, caspase-5 and caspase-11 can cleave GSDMD and release its pore-forming N-terminal fragment, which determines whether the cell enters a pyroptosis-like membrane rupture process (Shi et al., 2015). Another study using a nigericin-induced NLRP3 inflammasome model further confirmed this point through quantitative mass spectrometry and GSDMD gene deletion. GSDMD was required for pyroptosis and IL-1β secretion, but it was not required for the proteolytic maturation of IL-1β itself. This suggests that GSDMD acts more like an executor of release and membrane rupture, rather than an enzyme that processes inflammatory cytokines (He et al., 2015). Further *in vitro* membrane experiments and live-cell assays showed that the N-terminal fragment of GSDMD can directly target lipid membranes and form pores. Therefore, pyroptosis is not a passive collapse of the cell, but a lytic form of death actively caused by gasdermin-mediated changes in membrane structure (Sborgi et al., 2016). Compared with pyroptosis, the apoptotic branch is usually morphologically quieter, but in PANoptosis it does not occur as an isolated shrinkage-type death. Caspase-8, caspase-3 and caspase-7 can cleave apoptotic substrates, and they may also influence inflammasome activation and necroptosis-related molecules. Therefore, the apoptotic branch in PANoptosis functions more like an intermediate hub connecting inflammatory and non-inflammatory cell-death programs. This crosstalk is clearly illustrated by studies on GSDME. In some GSDME-positive cells, caspase-3 not only induces classical apoptosis, but also cleaves GSDME and releases the GSDME N-terminal fragment. As a result, a stimulus that would usually favor apoptosis can be converted into pyroptosis-like death with membrane rupture (Wang et al., 2017). The necroptotic branch is mainly associated with RIPK3 and MLKL. In general, activated RIPK3 promotes MLKL phosphorylation, after which MLKL translocates to membrane structures and disrupts membrane integrity, leading to leakage of intracellular contents and inflammatory amplification. However, in PANoptosis, the necroptotic branch does not always function in exactly the same way. For example, in IAV-induced ZBP1-related PANoptosis, caspases and RIPKs were identified as core components, but lytic cell death was not fully dependent on MLKL. This suggests that the three execution branches of PANoptosis show compensation and context dependence (Malireddi et al., 2023).

If PANoptosome assembly represents the integration of cell-death signaling, terminal membrane rupture represents the conversion of this death signal into tissue inflammatory injury. In the past, plasma membrane rupture after cell death was often regarded as a passive event, meaning that the cell simply collapsed after it had already died. Recent studies have changed this view. Ninjurin 1 (NINJ1) was identified as a key membrane protein that promotes plasma membrane rupture (PMR) during lytic cell death. Loss of NINJ1 markedly reduced lactate dehydrogenase release and the release of intracellular danger signals such as high mobility group box 1 (HMGB1) after pyroptosis, necrosis and apoptosis (Kayagaki et al., 2021). This finding indicates that the terminal stage of PANoptosis does not simply end after pore formation by GSDMD, GSDME or MLKL. NINJ1 may also be required for broader membrane rupture and intracellular content release. In other words, gasdermins and MLKL are important executors that initiate changes in membrane permeability, whereas NINJ1 may determine whether the cell proceeds to more complete lysis and inflammatory spread. In infection and heat-stress models, researchers further found that LPS combined with heat stress can induce PANoptosis dependent on caspase-1, caspase-11, caspase-8 and RIPK3, while NINJ1 mainly controls the release of inflammatory molecules and lethal changes *in vivo*. Deletion of NINJ1 reduced mortality in a mouse heat-stress model, and deletion of key PANoptosis molecules also reversed this pathological process (Han et al., 2024). Mechanistically, this process can be understood at two levels. At the first level, death-execution molecules generate pores or changes in membrane permeability. At the second level, terminal rupture molecules such as NINJ1 expand membrane damage and allow large amounts of inflammatory mediators and DAMPs to leak out. This two-step change may explain why some forms of cell death do not only reduce cell numbers, but also trigger persistent inflammation in surrounding tissues. Recent structural work further explained how NINJ1 mediates membrane rupture. Using cryo-electron microscopy, Researchers observed that NINJ1 can form oligomeric structures and proposed that NINJ1 mediates PMR by cutting and releasing membrane-disk-like structures. This provides more direct structural evidence that terminal membrane rupture is an actively regulated process (David et al., 2024).

**Figure 2:**
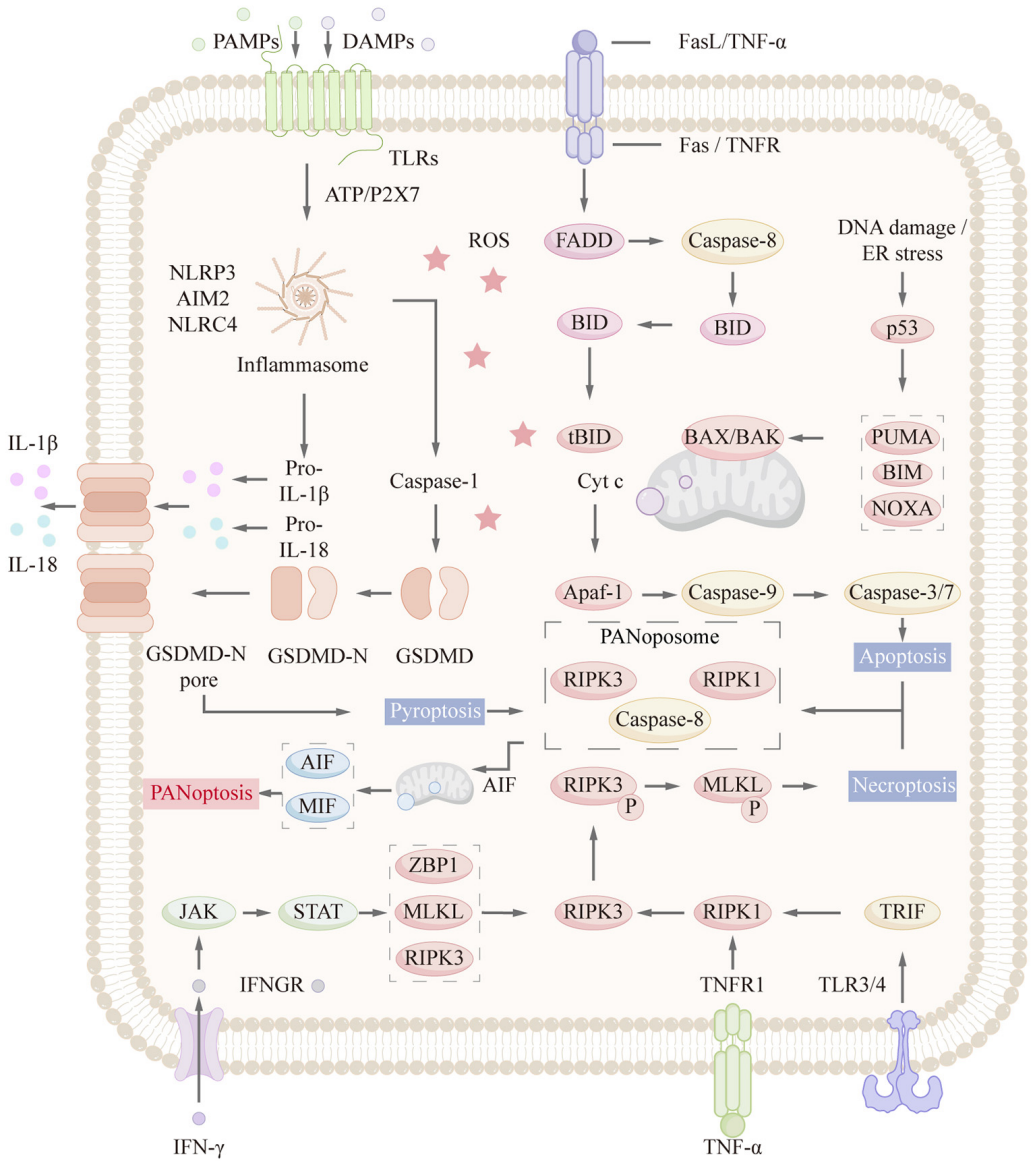
Integrated signaling network of pyroptosis, apoptosis, necroptosis, and PANoptosis. Pyroptosis, apoptosis, and necroptosis are interconnected forms of regulated cell death that can be activated by pathogen-associated molecular patterns (PAMPs), damage-associated molecular patterns (DAMPs), cytokine signaling, death receptor activation, mitochondrial stress, and DNA damage. PAMPs and DAMPs are sensed by pattern-recognition receptors, including Toll-like receptors (TLRs), and can promote inflammasome assembly through receptors such as NLRP3, AIM2, and NLRC4. Following inflammasome activation, caspase-1 cleaves pro-interleukin-1β (pro-IL-1β) and pro-interleukin-18 (pro-IL-18) into mature IL-1β and IL-18 and simultaneously cleaves gasdermin D (GSDMD), releasing the N-terminal fragment of GSDMD (GSDMD-N). GSDMD-N forms pores in the plasma membrane, enabling cytokine release and driving pyroptotic membrane permeabilization. In parallel, death receptor signaling initiated by Fas ligand (FasL) or tumor necrosis factor-α (TNF-α) activates Fas/TNFR-associated adaptor proteins, including FADD, which recruits and activates caspase-8. Caspase-8 can directly activate executioner caspases or cleave BID to truncated BID (tBID), thereby linking extrinsic apoptotic signaling to mitochondrial outer membrane permeabilization mediated by BAX and BAK. Mitochondrial cytochrome c release promotes apoptosome formation through Apaf-1 and caspase-9, resulting in caspase-3/7 activation and apoptotic cell death. DNA damage and endoplasmic reticulum stress further converge on p53-dependent transcriptional programs involving PUMA, BIM, and NOXA, thereby amplifying mitochondrial apoptosis. Necroptosis is primarily mediated by RIPK1, RIPK3, and mixed lineage kinase domain-like protein (MLKL). Upon TNFR1 or TLR3/4–TRIF signaling, RIPK1 and RIPK3 form necroptotic signaling complexes, leading to RIPK3-dependent MLKL phosphorylation, MLKL oligomerization, and membrane disruption. Interferon-γ (IFN-γ) signaling through the IFNGR–JAK–STAT pathway can induce ZBP1-associated signaling, further engaging RIPK3, MLKL, and PANoptosis-related cell death modules. Importantly, these death pathways do not operate independently. Caspase-8, RIPK1, RIPK3, FADD, inflammasome components, and mitochondrial mediators can assemble into PANoptosome-like complexes, coordinating the simultaneous activation of pyroptotic, apoptotic, and necroptotic machinery. In addition, mitochondrial release of apoptosis-inducing factor (AIF) and macrophage migration inhibitory factor (MIF) may contribute to PANoptosis-related DNA damage responses, adding another layer of crosstalk between mitochondrial injury and inflammatory cell death. Collectively, this network illustrates how inflammatory, mitochondrial, and death receptor signals converge to determine cell fate through context-dependent activation of pyroptosis, apoptosis, necroptosis, and PANoptosis. PAMPs, pathogen-associated molecular patterns; DAMPs, damage-associated molecular patterns; TLRs, Toll-like receptors; ATP, adenosine triphosphate; P2X7, purinergic receptor P2X7; NLRP3, NOD-like receptor family pyrin domain-containing 3; AIM2, absent in melanoma 2; NLRC4, NOD-like receptor family CARD domain-containing 4; IL-1β, interleukin-1β; IL-18, interleukin-18; GSDMD, gasdermin D; GSDMD-N, N-terminal fragment of gasdermin D; ROS, reactive oxygen species; FasL, Fas ligand; TNF-α, tumor necrosis factor-α; TNFR, tumor necrosis factor receptor; FADD, Fas-associated death domain protein; BID, BH3-interacting domain death agonist; tBID, truncated BID; BAX, BCL2-associated X protein; BAK, BCL2 antagonist/killer; Cyt c, cytochrome c; Apaf-1, apoptotic protease-activating factor 1; PUMA, p53 upregulated modulator of apoptosis; BIM, BCL2-like 11; NOXA, phorbol-12-myristate-13-acetate-induced protein 1; RIPK1, receptor-interacting serine/threonine-protein kinase 1; RIPK3, receptor-interacting serine/threonine-protein kinase 3; MLKL, mixed lineage kinase domain-like protein; TRIF, TIR-domain-containing adapter-inducing interferon-β; IFN-γ, interferon-γ; IFNGR, interferon-γ receptor; JAK, Janus kinase; STAT, signal transducer and activator of transcription; ZBP1, Z-DNA-binding protein 1; AIF, apoptosis-inducing factor; MIF, macrophage migration inhibitory factor.

Overall, the mechanism of PANoptosis can be summarized in four connected steps. Cells first recognize danger signals generated by infection, hemorrhage, metabolic disturbance or tissue injury. Then, sensors such as ZBP1, AIM2, NLRP12, NLRC5 or NLRP3 participate in PANoptosome assembly. Next, execution molecules such as caspase-1, caspase-8, GSDMD, RIPK3 and MLKL are activated in parallel. Finally, membrane rupture and inflammatory mediator release amplify injury in the surrounding tissue. For neurological diseases, the value of this mechanism is that it explains why markers of pyroptosis, apoptosis and necroptosis often appear together. It also reminds researchers that therapeutic strategies should not focus only on one molecule, but should consider combined regulation of upstream inflammatory signals, death-complex assembly and terminal membrane rupture (Figure 2).

## 4 Mechanism of PANoptosis in neurological diseases

### 4.1 AD

In AD, PANoptosis should not be understood as a theory replacing the amyloid-beta (Aβ) or tau hypotheses, but as an amplifying link that connects protein deposition, microglial inflammation, and neuronal death. In APP/PS1 mice and brain tissues from patients with mild cognitive impairment and AD, Heneka and colleagues found activation of the NLRP3 inflammasome. Genetic deletion of *Nlrp3* or *Casp1* reduced spatial memory impairment, decreased IL-1β, and enhanced Aβ clearance, suggesting that inflammasome activation can drive AD progression (Heneka et al., 2013). This finding mainly supports a pyroptosis-related mechanism. However, it is also relevant to PANoptosis. NLRP3 activation, caspase-1 activity, cytokine release, and cell lysis can maintain local inflammation. Under this inflammatory condition, apoptosis and necroptosis may be more easily activated. Venegas and colleagues further studied ASC specks released from microglia. They found that these ASC specks could bind directly to Aβ and promote Aβ aggregation *in vitro*. In APP/PSEN1 mice, intrahippocampal injection of ASC specks aggravated Aβ pathology. This effect was reduced when an anti-ASC antibody was used (Venegas et al., 2017). This suggests that inflammasomes in AD are not limited to cytokine production. ASC specks may also link cell death-related signals with protein aggregation. In this way, inflammatory injury may contribute to the spread of pathological changes in brain tissue. Tau normally contributes to the stability of axonal microtubules. In AD brains, this function is disrupted when tau becomes abnormally phosphorylated. Phosphorylated tau can dissociate from microtubules and progressively assemble into paired helical filaments and neurofibrillary tangles (Grundke-Iqbal et al., 1986). Ising and colleagues studied this issue using tau transgenic models and Aβ-related models. Their results showed that activation of the NLRP3 inflammasome increased tau hyperphosphorylation and aggregation. This finding suggests that Aβ-induced microglial inflammation may help connect Aβ pathology with tau pathology (Ising et al., 2019). Necroptosis may also contribute to AD pathology. Caccamo and colleagues reported increased RIPK1 signaling in postmortem brain tissues from patients with AD. They also found that necroptosis-related signals were associated with a higher Braak stage, lower brain weight, and worse cognitive performance (Caccamo et al., 2017). Salvadores and colleagues provided more direct experimental evidence for this process. They found that conditioned medium from Aβ oligomer-stimulated microglia triggered neuronal necroptosis through TNF-α signaling. Blocking necroptosis reduced neuronal injury. This finding indicates that inflammatory cells may affect the death mode of neighboring neurons (Salvadores et al., 2022). In AD, PANoptosis should be described as a coordinated but complex process. Aβ and tau pathology can activate microglial inflammasomes. Inflammatory cytokines, ASC specks, TNF-α, and RIPK1-related signaling may further connect pyroptosis, apoptosis, and necroptosis, allowing these death pathways to amplify one another. Recent studies using bulk RNA sequencing and single-nucleus RNA sequencing have also found links between PANoptosis-related genes and immune dysregulation in AD brains. However, most of this evidence is still based on computational analysis. Further cellular and animal studies are needed to determine whether these key molecules can truly form a PANoptosome (Liu et al., 2025).

### 4.2 PD

In PD, discussion of PANoptosis mainly centers on α-synuclein (α-syn) aggregation, microglial inflammation, and dopaminergic (DA) neuronal death. In the study by Gordon and colleagues, ASC and cleaved caspase-1 were increased in the substantia nigra of PD patients and in multiple PD models. The authors further showed that fibrillar α-syn could activate the NLRP3 inflammasome in microglia. Importantly, MCC950, an NLRP3 inhibitor, alleviated motor deficits, reduced DA neuronal loss, and decreased α-syn pathology (Gordon et al., 2018). Pike and colleagues further showed in primary human microglia that α-syn fibrils induced NLRP3-dependent IL-1β secretion, and super-resolution microscopy revealed recruitment of caspase-1 to inflammasome scaffolds, giving more human-relevant evidence that microglia can sense α-syn and generate inflammatory death signals in PD (Pike et al., 2021). In a human study, Fan and colleagues measured peripheral NLRP3 inflammasome activation and plasma α-syn levels in patients with PD, and found that these markers correlated with motor severity and disease progression, suggesting that inflammasome activation is not only a model phenomenon but may also reflect systemic inflammation in patients (Fan et al., 2020). Furthermore, in an AAV1/2-A53T α-syn progressive PD mouse model, Grotemeyer and colleagues found that MCC950 not only protected DA neurons, but also reduced microglial activation and CD4-positive and CD8-positive T-cell infiltration, indicating that NLRP3 may connect neuronal protein pathology with innate and adaptive immunity (Grotemeyer et al., 2023). Beyond pyroptosis-like inflammation, necroptosis is also an important death mode in PD, as Oñate and colleagues found activation of the necroptosis machinery in PD patient brain tissue and toxin-induced models, and inhibition of MLKL, RIPK3, or RIPK1 reduced axonal degeneration and motor deficits (Oñate et al., 2020). Another MPTP mouse study showed that increased RIPK1/RIPK3/MLKL proteins were associated with DA neuronal loss, while Nec-1 or deficiency of RIPK3 or MLKL preserved dopamine content and neuronal number, indicating that necroptosis in PD is not merely an accompanying event but may directly contribute to neurodegeneration (Lin et al., 2020). A recent Parkin-focused study more directly linked PD with PANoptosis, as the authors found coexistence of PANoptosis and DA neuronal injury in rotenone-treated SN4741 cells and mice, while Parkin promoted NLRP3 degradation through chaperone-mediated autophagy and thereby suppressed PANoptosis (Zheng et al., 2026). Therefore, PANoptosis in PD can be summarized as a crosstalk network among α-syn-induced inflammasome activation, RIPK1/RIPK3/MLKL-mediated necroptosis, and DA neuronal apoptosis, with NLRP3 and RIPK1 likely serving as central convergence nodes.

### 4.3 IS

Cell death after IS shows clear temporal and spatial heterogeneity, with rapid necrosis more prominent in the ischemic core, while inflammation, apoptosis, pyroptosis, and necroptosis are more likely to overlap in the penumbra and reperfused regions. Lan and colleagues directly studied neuronal PANoptosis after cerebral ischemia/reperfusion injury, and found that curcumin-primed olfactory mucosa-derived mesenchymal stem cells reduced neuronal PANoptosis and ischemia/reperfusion injury by modulating microglial polarization, providing relatively direct animal and cellular evidence that PANoptosis occurs in IS (Lan et al., 2024). Using a middle cerebral artery occlusion (MCAO) model, Zhang et al. discovered an accumulation of insoluble RIPK1/RIPK3/MLKL complexes within the ischemic region. They found that RIPK1 kinase inactivation, RIPK3 deficiency, or MLKL deficiency each reduced infarct volume and alleviated neuroinflammation, suggesting the occurrence of necroptosis (Zhang et al., 2020). Deng and colleagues found in a rat IS model that Nec-1 reduced brain necroptosis by inhibiting RIPK1-mediated RIPK3/MLKL signaling, indicating that RIPK1 is not only an upstream kinase for death execution but may also be an entry point into the post-stroke inflammatory death network (Deng et al., 2019). Furthermore, pyroptosis-related work also supports this crosstalk network, as Kim and colleagues found that absent in AIM2 inflammasome increased in the hippocampus and cortex after transient MCAO in mice and was associated with caspase-1, IL-1β, and IL-18 upregulation, while AIM2 deletion or caspase-1 inhibition improved chronic post-stroke cognitive impairment and brain atrophy (Kim et al., 2020). A study of Nicorandil further showed that in oxygen-glucose deprivation/reoxygenation-treated BV2 microglia and in an *in vivo* stroke model, inhibition of the nuclear factor-κB (NF-κB)/AIM2/GSDMD pathway reduced inflammatory and pyroptotic markers and improved post-stroke neurological function, suggesting that AIM2 may convert ischemic stress into inflammatory cell death (Zhao et al., 2024). The Tangeretin study added an antioxidant perspective to this chain, showing that it inhibited AIM2 inflammasome activation and reduced cerebral ischemia/reperfusion-induced neuronal pyroptosis by regulating nuclear factor erythroid 2-related factor 2 (Nrf2), while the Nrf2 inhibitor ML385 weakened this protection (You et al., 2024). Therefore, in IS, PANoptosis explains why the inhibition of a single death pathway often provides limited protection, as inflammasomes, RIPK1/RIPK3/MLKL, mitochondrial damage, oxidative stress, and neuronal apoptosis can interact with one another within the same lesion following reperfusion.

### 4.4 SAH

After SAH, hemoglobin, heme, iron ions, and oxidative stress rapidly stimulate meningeal, vascular, and parenchymal brain cells, making inflammatory cell death an important component of early brain injury. The link between SAH and PANoptosis is currently more direct than in many chronic neurodegenerative diseases, because a phosphoglycerate mutase family member 5 (PGAM5) study showed that PGAM5 translocated to mitochondria-free cytosolic fractions after SAH and promoted RIPK1-PANoptosome activity by phosphorylating and activating RIPK1, in that rat SAH model, reducing PGAM5 decreased PANoptosis and improved both short-term and long-term neurological function, suggesting that the PGAM5-RIPK1 axis may act as a key switch for the combined activation of multiple death programs after SAH (Jiajia et al., 2025). NLRP3 is also an important node of inflammatory death after SAH, as Dodd and colleagues found that NLRP3 inhibition attenuated early brain injury and delayed cerebral vasospasm, suggesting that inflammasome activation can influence both acute neural injury and delayed vascular complications (Dodd et al., 2021). In patients with aneurysmal SAH, investigators examined monocytes and plasma and found NLRP3 overactivation in monocytes, together with increased plasma IL-1β, IL-18, GSDMD, and tissue factor, indicating that post-SAH inflammasome activation is not limited to animal models but can also be observed in human peripheral immunity (Díaz-García et al., 2023). The necroptosis pathway can also feed back onto inflammasome activation, as a rat SAH study showed that the RIPK1-RIPK3-dynamin-related protein 1 (DRP1) pathway regulated NLRP3 inflammasome activation, suggesting a continuous reaction among mitochondrial injury, necroptosis, and pyroptosis (Zhou et al., 2017). Another *in vivo* and *in vitro* SAH study found that RIPK1 kinase activation was associated with both microglial inflammation and neuronal apoptosis, and RIPK1 inhibition reduced neuroinflammation and neuronal injury, mechanistically supporting RIPK1 as a hub linking necroptosis, inflammation, and apoptosis (Wu et al., 2024). RIPK3 work supports the same idea, as inhibition of RIPK3 by GSK'872 attenuated early brain injury after SAH, while RIPK3 expression began to increase at 6 h after hemorrhage, peaked at 72 h, and was mainly observed in neurons (Chen et al., 2018). Based on this, PANoptosis in SAH can be summarized as a process in which blood degradation products and oxidative stress first activate NLRP3 and mitochondrial injury, and then molecules such as RIPK1, RIPK3, DRP1, and PGAM5 integrate pyroptosis, apoptosis, and necroptosis into a shared pathway that aggravates early brain injury.

### 4.5 ICH

Secondary injury after ICH is not caused only by mechanical compression from the hematoma, because hemoglobin degradation, iron release, oxidative stress and inflammatory mediators together create an environment that can activate several PCD pathways (Keep et al., 2012; Lule et al., 2017). From the perspective of PANoptosis, ICH is characterized by the coexistence of pyroptosis, apoptosis and necroptosis in the perihematomal region, so it is viewed as a multi-pathway injury network (Lule et al., 2021). In the pyroptotic branch, the HMGB1 and TLR4 axis can activate the NLRP3 inflammasome, which then promotes caspase-1 and GSDMD-mediated membrane pore formation and increases the release of inflammatory cytokines such as IL-1β (Lei et al., 2024). Furthermore, researchers utilized *in vivo* ICH models and *in vitro* microglial models to discover that ursolic acid can reduce the levels of phosphorylated NF-κB, GSDMD-N, cleaved caspase-1, TNF-α, IL-6, and IL-1β. Moreover, reactivating NF-κB with phorbol 12-myristate 13-acetate was found to diminish this protective effect, suggesting that pyroptosis not only kills the cells themselves but also transforms cell death into new inflammatory signals, thereby continuing to recruit microglia and peripheral immune cells into the site of injury (Lei et al., 2023). In the necroptotic branch, the signaling axis formed by RIPK1, RIPK3 and MLKL provides an important basis for membrane rupture-like neuronal death after ICH (Lule et al., 2017). A mouse ICH study further showed that RIPK3 activation promoted death domain-associated protein 6 (DAXX)-dependent neuronal necroptosis, and the researchers used ribonucleic acid sequencing, quantitative polymerase chain reaction, enzyme-linked immunosorbent assay, immunofluorescence and co-immunoprecipitation to show that DAXX was increased after ICH in both patients and mice, while inhibition of DAXX or RIPK3 reduced brain edema and neurological deficits (Bai et al., 2024). This necroptotic signal also has clinical relevance, because a prospective cohort study measured serum RIPK3 in 183 patients with ICH and 100 controls, and found that higher RIPK3 levels were associated with early hematoma growth and poor neurological outcomes (Li et al., 2024). At the same time, both an *in vivo* autologous blood injection model and an *in vitro* oxidized hemoglobin stimulation model suggested that GATA binding protein 4 participates in neuronal apoptosis through the p53 pathway (Xu et al., 2019). Another animal study showed that glibenclamide reduced activated caspase-3 and increased the ratio of Bcl-2 to Bcl-2-associated X protein (Bax), indicating that regulation of the mitochondrial apoptotic pathway may reduce neurological injury after ICH (Zhou et al., 2018).

### 4.6 HD

HD is an inherited neurodegenerative disease caused by mutant huntingtin (mHtt), and the most vulnerable cells are striatal medium spiny neurons, although interactions among inflammation, energy metabolism dysfunction and cell death also contribute to disease progression (Paldino et al., 2020b). In the R6/2 mouse model, researchers examined brain tissue at 4 and 13 weeks of age and found that NLRP3 and caspase-1 were markedly increased at 13 weeks, especially in vulnerable striatal spiny projection neurons and parvalbumin-positive interneurons (Paldino et al., 2020b). These findings indicate that inflammasome activation in HD may not simply be a bystander response. Instead, it can occur within vulnerable neuronal populations and may connect protein aggregation with inflammation and cell death. Recent work has also expanded the study of pyroptosis beyond neurons. In brain microvascular endothelial cells, mHtt was shown to activate the NLRP3 inflammasome. This process may promote endothelial pyroptosis, blood-brain barrier disruption, and secondary neuronal injury (Cai et al., 2025). This finding helps explain how endothelial inflammation and barrier dysfunction may allow peripheral inflammatory signals to influence the brain environment more easily. In an intervention experiment, the poly(ADP-ribose) polymerase 1 (PARP-1) inhibitor olaparib improved survival and neurobehavioral performance in R6/2 mice, while reducing inflammasome activation and pyroptosis-related and apoptosis-related changes in the striatum (Paldino et al., 2020a). Another study treated R6/2 mice with the selective NLRP3 inhibitor MCC950 and showed that it increased neuronal survival, reduced neuroinflammation, prolonged lifespan and improved motor function, further supporting the inflammasome pathway as an intervention target in HD (Chen et al., 2022). In the apoptotic branch, earlier animal studies found that the Bcl-2 family proteins Bid and BimEL have distinct roles in neuronal dysfunction in HD mouse models, indicating that mitochondrial apoptotic regulation is not merely a late-stage consequence of the disease (García-Martínez et al., 2007). In R6/1 mice, increased Bax expression and more terminal deoxynucleotidyl terminal deoxynucleotidyl transferase dUTP nick-end labeling (TUNEL)-positive cells were associated with disease progression, especially in regions such as the cortex, indicating that pro-apoptotic stress accumulates as the disease advances (Teles et al., 2008). Wild-type huntingtin has also been shown to exert anti-apoptotic effects upstream of caspase-3, which means that mHtt may not only gain toxic functions but also weaken the normal survival-supporting function of huntingtin (Rigamonti et al., 2000).

### 4.7 SCI

The pathological process after SCI is usually divided into primary mechanical injury and secondary injury, and the secondary injury includes inflammatory spread, disruption of the blood-spinal cord barrier, oxidative stress, excitotoxicity and multiple forms of cell death (Liu et al., 2018). This secondary injury cascade fits the PANoptosis framework well, because microglial pyroptosis, neuronal apoptosis and RIPK1/RIPK3/MLKL-related necroptosis may coexist in the same injured tissue. In the pyroptotic branch, a cluster of differentiation 73 (CD73) study combined patient blood samples, animal models and cellular models, and showed that CD73 inhibited GSDMD maturation and NLRP3 inflammasome activation through the adenosine A2B receptor, phosphoinositide 3-kinase (PI3K)/protein kinase B (AKT)/forkhead box O1 (FOXO1) pathway, thereby reducing microglial pyroptosis (Xu et al., 2021). Butyrate-related research also supports that gut-derived metabolites can influence inflammatory cell death after SCI, because high-fiber diet or intrathecal butyrate inhibited histone deacetylase 1 and the NLRP3 inflammasome in a C5 hemicontusion model and in microglia stimulated by LPS plus adenosine triphosphate, while improving oxidative stress and functional recovery (Ganggang et al., 2025). In an earlier sodium butyrate study, researchers used a mouse SCI model and found that sodium butyrate improved histological injury and motor function, while reducing NF-κB pathway-related inflammatory cytokines such as IL-1β and TNF-α (Lanza et al., 2019). In the necroptotic branch, lysosomal damage after SCI can cause the accumulation of RIPK1 and RIPK3 proteins and enhance MLKL-related necroptotic signaling, and researchers also reproduced this process in PC12 cells and primary neurons using lysosomal inhibitors. This study also showed that rapamycin, which improves autophagic and lysosomal function, reduced RIPK1 accumulation and cell death, suggesting that necroptosis does not occur in isolation but may be linked to impaired clearance of damaged proteins and organelles (Liu et al., 2018). In the apoptotic branch, Bcl-2-overexpressing mice showed less neuronal apoptosis, smaller lesion size and better behavioral recovery after T10 contusive SCI, indicating that strengthening anti-apoptotic capacity can reduce secondary neural injury (Seki et al., 2003; Wang et al., 2011). Another study showed that miR-222-3p reduced neuronal apoptosis by targeting Bax, and decreased caspase-3, caspase-9 and cytochrome c-related changes in both a rat SCI model and a hypoxic neuronal model (Seki et al., 2003; Zhang et al., 2023). In addition, a rat study of acute SCI measured markers such as microtubule-associated protein 1 light chain 3, activated caspase-3, Bcl-2 and Bax, suggesting a link between autophagic changes and apoptosis, which further indicates that the post-SCI cell death network is highly interconnected (Hou et al., 2014).

### 4.8 ALS

ALS is characterized mainly by progressive loss of motor neurons, but the pathology is not limited to motor neurons themselves, because microglia, astrocytes and peripheral immune responses also influence disease progression (Johann et al., 2015). In microglial pyroptosis, researchers used Nlrp3-GFP knock-in mice and primary microglia and found that both aggregated and soluble superoxide dismutase 1 (SOD1)-G93A proteins activated NLRP3, promoted ASC speck formation, caspase-1 activation and IL-1β release (Deora et al., 2020). This result indicates that ALS-related misfolded proteins are not only toxic burdens inside neurons, but can also be recognized by microglia as danger signals, thereby converting proteostasis stress into inflammatory cell death signaling. TAR DNA-binding protein 43 can also activate microglia through CD14, and further activate NF-κB, activator protein 1 and the NLRP3 inflammasome, thereby increasing IL-1β production (Zhao et al., 2015; Yuan et al., 2025). In astrocytes, NLRP3 inflammasome components and IL-1β were detected in both the SOD1 mouse model and postmortem tissue from patients with sporadic ALS. Notably, these changes appeared before symptom onset. This suggests that glial inflammation may be involved in the early stage of disease progression (Johann et al., 2015). Evidence from wobbler mice further supports this view. In this model, spinal cord levels of NLRP3, pro-caspase-1, cleaved caspase-1, full-length GSDMD, and cleaved GSDMD were increased. These signals were found together with neurons, microglia, and astrocytes (Cihankaya et al., 2024). These results indicate that inflammasome responses in ALS are unlikely to be restricted to a single cell type. Instead, inflammatory signaling between neurons and glial cells may create a self-amplifying injury loop. In the apoptotic branch, caspase-1 and caspase-3 can be sequentially activated in mutant SOD1 mice, while Bcl-2 overexpression delays caspase activation and disease progression, suggesting a temporal link between apoptosis-like signaling and motor neuron death (Vukosavic et al., 2000). In the necroptotic branch, RIPK1 has been shown to contribute to ALS-related axonal degeneration by promoting inflammation and necroptosis, and researchers observed RIPK1 pathway-related changes in optineurin-deficient models, SOD1-G93A mice and samples from patients with ALS (Ito et al., 2016). From a translational perspective (Wei et al., 2023), RIPK1 remains worth attention, because one study found that primidone delayed disease onset and progression in SOD1-G93A mice, and observed that serum RIPK1 and IL-8 were increased in 162 ALS participants and decreased after treatment (Table 1).

**Table 1:** Revised evidence map of PANoptosis-related programmed cell death in neurological disorders.

Disease types	Models	Cell types	PCD	Markers	Direct/ indirect	PANoptosome-related evidence	Representative interventions	Evidence limitations	References
AD	C57BL/6 mice	Microglia	Pyroptosis	NLRP3, caspase-1, IL-1β	Indirect	NLRP3/Casp1 loss links inflammasome activation to AD injury	Nlrp3 or Casp1 genetic deletion in APP/PS1 mice	Inflammasome-focused; lacks caspase-3/ RIPK-MLKL co-profiling	Heneka et al., 2013
	C57BL/6J mice	Microglia	Pyroptosis	ASC specks, NLRP3, ASC, IL-1β	Indirect	ASC specks propagate Aβ-associated inflammasome signaling	Anti-ASC antibody; ASC deficiency; intrahippocampal ASC-speck seeding paradigm	Inflammasome propagation only; lacks apoptosis/ necroptosis profiling	Venegas et al., 2017
	C57BL/6 mice	Microglia	Pyroptosis	NLRP3, ASC, caspase-1, IL-1β	Indirect	NLRP3 loss attenuates tau pathology and inflammatory injury	Nlrp3 deficiency or inflammasome-function loss in tau-transgenic mice	Inflammasome crosstalk only; lacks RIPK/MLKL and caspase-3 data	Ising et al., 2019
	C57BL/6J mice	Hippocampal neurons	Pyroptosis	Caspase-1, IL-1β	Indirect	Caspase-1 blockade improves cognition and pathology	VX-765 pharmacological caspase-1 inhibition; Casp1 genetic ablation	Strong outcomes; lacks parallel apoptosis/ necroptosis profiling	Flores et al., 2018
PD	C57BL/6 mice	Microglia	Pyroptosis	NLRP3, ASC, caspase-1, IL-1β	Indirect	α-Syn activates NLRP3; inhibition protects DA neurons	MCC950 NLRP3 inhibition; Nlrp3 deficiency	Robust inflammasome data; lacks RIPK and apoptosis integration	Gordon et al., 2018
	C57BL/6J mice	Microglia	Pyroptosis	NLRP3, IL-1β, caspase-1	Indirect	MCC950 supports inflammasome-driven PD injury	MCC950 inflammasome/NLRP3 inhibition	Inflammasome-focused; lacks RIPK/MLKL and complex assays	Grotemeyer et al., 2023
	C57BL/6 mice	Dopaminergic neurons	Necroptosis	RIP1, RIP3, MLKL	Indirect	RIP1/RIP3/MLKL activation marks PD necroptotic injury	Necroptosis-pathway inhibition/assessment in MPTP mice	Necroptosis-focused; lacks NLRP3/GSDMD and caspase-3 profiling	Lin et al., 2020
	C57BL/6 mice	Dopaminergic neurons	Pyroptosis	NLRP3, caspase-1, IL-1β	Indirect	Echinacoside suppresses NLRP3 signaling and preserves DA neurons	Echinacoside administration	Pharmacological inflammasome evidence; lacks necroptosis/complex assays	Gao et al., 2020
	C57BL/6 mice	Dopaminergic neurons	PANoptosis	NLRP3, caspase-1, IL-1β	Direct	Parkin/CMA promotes NLRP3 degradation and suppresses PANoptosis	Parkin overexpression; CMA activation; NLRP3 inhibition/ degradation targeting	Recent mechanistic study; needs replication and spatial validation	Zheng et al., 2026
IS	C57BL/6 mice	Neurons	PANoptosis	NLRP3, GSDMD, caspase-1, cleaved caspase-3, Bax, Bcl-2, RIPK1, RIPK3, MLKL	Direct	CUR-OM-MSCs reduce coordinated neuronal PANoptosis markers	Curcumin-primed olfactory mucosa-derived mesenchymal stem cells (CUR-OM-MSCs)	PANoptosis-relevant; complex assembly needs spatial/proteomic validation	Lan et al., 2024
IS	C57BL/6 mice	Neurons	Necroptosis	RIP1, RIP3, MLKL, p-MLKL	Indirect	RIP1/RIP3/MLKL genetics show necroptosis-driven stroke injury	RIP1 kinase inactivation; Rip3 or Mlkl deficiency	Strong necroptosis genetics; lacks pyroptosis/ apoptosis co-profiling	Zhang et al., 2020
	Sprague-Dawley rats	Neurons	Necroptosis	p-RIPK1, RIPK3, MLKL, p-MLKL, mature IL-1β	Indirect	Nec-1 reduces RIPK1/RIPK3/MLKL and IL-1β after ischemia	Necrostatin-1 RIPK1 inhibition	Necroptosis well characterized; lacks caspase-3/ GSDMD profiling	Deng et al., 2019
	C57BL/6 mice	Microglia	Pyroptosis	AIM2, ASC, caspase-1, IL-1β, IL-18	Indirect	AIM2 inflammasome amplifies post-stroke injury	AIM2 genetic deletion/deficiency	Inflammasome evidence; needs RIPK and apoptosis colocalization	Kim et al., 2020
	C57BL/6 mice	Neurons	Pyroptosis	NLRP3, ASC, caspase-1, IL-1β	Indirect	MCC950 links NLRP3 inhibition to neuroprotection	MCC950 NLRP3 inflammasome inhibition	Therapeutic inflammasome study; lacks necroptosis/ interaction assays	Ismael et al., 2018
SAH	Sprague-Dawley rats	Neurons	PANoptosis	NLRP3, caspase-1, GSDMD, cleaved caspase-3, RIPK1, RIPK3, MLKL, PGAM5	Direct	PGAM5 activates RIPK1-PANoptosome after SAH	PGAM5 reduction/ manipulation; RIPK1-PANoptosome pathway targeting	Strong PANoptosome evidence; needs cross-species/ human validation	Jiajia et al., 2025
	C57BL/6J mice	Neurons	Pyroptosis	NLRP3, IL-1β	Indirect	NLRP3 inhibition reduces EBI, vasospasm and apoptosis	MCC950 NLRP3 inhibition	Pyroptosis-apoptosis evidence; lacks RIPK and complex assays	Dodd et al., 2021
	Sprague-Dawley rats	Neurons	Necroptosis	NLRP3, RIP1, RIP3, phosphorylated DRP1	Indirect	RIP1/RIP3/DRP1 regulates NLRP3 activation	Necrostatin-1 RIP1 inhibition	Strong necroptosis-inflammasome crosstalk; lacks apoptosis validation	Zhou et al., 2017
	Sprague-Dawley rats	Neurons	Necroptosis	RIPK3, MLKL, HMGB1	Indirect	RIPK3 blockade reduces MLKL-associated SAH injury	GSK'872 RIPK3 inhibition	Necroptosis-focused; lacks inflammasome/ apoptosis integration	Chen et al., 2018
ICH	C57BL/6 mice	Microglia/ macrophages	Pyroptosis	HMGB1, TLR4, NLRP3, caspase-1, GSDMD, IL-1β	Indirect	HMGB1/TLR4 induces NLRP3/GSDMD pyroptosis after ICH	HMGB1/TLR4 pathway blockade or modulation in ICH setting	Pyroptosis-focused; lacks apoptosis/ necroptosis validation	Lei et al., 2024
	Sprague-Dawley rats	Microglia/ macrophages	Pyroptosis	NF-κB, NLRP3, GSDMD-N, cleaved caspase-1, IL-1β, IL-6, TNF-α	Indirect	Ursolic acid suppresses NF-κB/NLRP3/ GSDMD pyroptosis	Ursolic acid; PMA pathway reactivation for mechanism testing	Microglial pyroptosis pharmacology; lacks apoptosis/ necroptosis coordination	Lei et al., 2023
	C57BL/6 mice	Neurons	Necroptosis	RIPK1, MLKL	Indirect	Cell-specific RIPK1/ MLKL activation defines ICH necroptosis	RIPK1 kinase-dead genetic inhibition	Cell-specific necroptosis mapping; lacks inflammasome/ apoptosis integration	Lule et al., 2021
	C57BL/6 mice	Neurons	Necroptosis	RIPK3, DAXX, MLKL	Indirect	RIPK3-DAXX-MLKL drives neuronal necroptosis	RIPK3/DAXX pathway inhibition or genetic/ pharmacological manipulation	Necroptosis-specific; lacks inflammasome and apoptotic testing	Bai et al., 2024
	C57BL/6 mice	Microglia/ macrophages	Pyroptosis	NLRP3, caspase-1, IL-1β, IL-18	Indirect	MCC950 interrupts NLRP3-dependent ICH injury	MCC950 selective NLRP3 inflammasome inhibition	Inflammatory injury data; lacks RIPK/MLKL and interaction assays	Ren et al., 2018
HD	B6CBA-Tg(HDexon1)62Gpb mice	Striatal neurons	Pyroptosis	Caspase-1, IL-1β	Indirect	Caspase-1/IL-1β activation tracks HD progression	Disease-stage comparison; no pharmacological intervention in this specific study	Branch-level evidence; lacks intervention and necroptosis data	Paldino et al., 2020b
	B6CBA-Tg(HDexon1)62Gpb mice	Striatal neurons	Pyroptosis	NLRP3, caspase-1, IL-1β	Indirect	Olaparib modulates inflammasome/pyroptosis in HD	Olaparib, a PARP-1 inhibitor	Pyroptosis therapy evidence; lacks necroptosis/ interaction assays	Paldino et al., 2020a
	B6CBA-Tg(HDexon1)62Gpb mice	Striatal neurons	Pyroptosis	ASC, caspase-1, IL-1β, NLRP3	Indirect	MCC950 suppresses NLRP3 signaling and improves HD readouts	MCC950 NLRP3 inflammasome inhibition	Inflammasome-targeted; lacks RIPK/MLKL and cell-specific colocalization	Chen et al., 2022
	Wistar rats	Striatal neurons	Pyroptosis	NLRP3	Indirect	Parthenolide inhibits NLRP3-linked HD-like injury	Parthenolide treatment	HD model useful; lacks necroptosis testing	Noureldeen et al., 2024
SCI	C57BL/6J mice	Microglia/ macrophages	Pyroptosis	NLRP3, GSDMD, GSDMD-N, caspase-1	Indirect	CD73 signaling suppresses GSDMD-mediated microglial pyroptosis	CD73 genetic manipulation; A2B/PI3K/AKT/Foxo1 pathway modulation	Microglial pyroptosis mechanism; lacks necroptosis/complex assays	Xu et al., 2021
	Sprague-Dawley rats	Microglia/ macrophages	Pyroptosis	HDAC1, NLRP3	Indirect	Butyrate inhibits HDAC1/NLRP3-mediated inflammation	High-fiber diet-derived butyrate; HDAC1/NLRP3 targeting	Recent animal data; needs replication and branch integration	Ganggang et al., 2025
	CD1 mice	Neurons	Pyroptosis	NF-κB, IL-1β, TNF-α	Indirect	Sodium butyrate suppresses inflammatory injury signaling	Sodium butyrate administration	Anti-inflammatory evidence; lacks canonical inflammasome/ necroptosis profiling	Lanza et al., 2019
	C57BL/6 mice	Neurons	Necroptosis	LC3-II, p62/SQSTM1, RIPK1, RIPK3, MLKL	Indirect	Lysosomal damage promotes RIPK1/RIPK3/ MLKL necroptosis	Rapamycin stimulation of autophagy/lysosomal function	Autophagy-necroptosis link; lacks NLRP3/GSDMD and caspase-3 data	Liu et al., 2018
	C57BL/6 mice	Microglia/ macrophages	Pyroptosis	NLRP3, IL-1β, IL-18	Indirect	OLT1177 reduces NLRP3-dependent SCI inflammation	OLT1177/ dapansutrile NLRP3 inhibition	Inflammasome-targeted; lacks necroptosis and complex assays	Amo-Aparicio et al., 2022
	C57BL/6 mice	Microglia/ macrophages	Pyroptosis	Caspase-1, IL-1β, IL-18	Indirect	VX-765 reduces caspase-1-dependent neuroinflammation	VX-765 caspase-1 inhibition for 7 days after SCI	Caspase-1 evidence; lacks RIPK and PANoptosome analyses	Chen et al., 2021
ALS	B6SJL-Tg(SOD1^*^G93A)1Gur/J mice	Microglia	Pyroptosis	NLRP3, ASC, caspase-1, IL-1β	Indirect	ALS proteins activate microglial NLRP3 inflammasome	ALS-protein stimulation; inflammasome-pathway inhibition/ assessment in microglial systems	Microglial inflammasome evidence; lacks *in vivo* PANoptosis integration	Deora et al., 2020
	B6SJL-Tg(SOD1^*^G93A)1Gur/J mice	Astrocytes	Pyroptosis	NLRP3, ASC, caspase-1	Indirect	Astrocytic NLRP3 expression indicates glial inflammasome activation	Disease-stage/ cell-type comparison; therapeutic intervention was not a central component	Localization evidence; lacks intervention and apoptosis/necroptosis markers	Johann et al., 2015
	B6SJL mice	Spinal motor neurons	Apoptosis	Caspase-1, caspase-3, β-actin, Bcl-2	Indirect	Caspase-1-to-caspase-3 sequence supports apoptosis coupling	Bcl-2 overexpression in transgenic mSOD1 mice	Apoptosis-caspase crosstalk; lacks RIPK/PANoptosome testing	Vukosavic et al., 2000
	C57BL/6 mice	Motor axons	Necroptosis	RIPK1, RIPK3, MLKL	Indirect	RIPK1/RIPK3/ MLKL mediates axonal necroptosis/ inflammation	RIPK1 kinase inhibition; Ripk3 deficiency/ genetic modulation	Strong necroptosis evidence; lacks inflammasome/ apoptosis co-mapping	Ito et al., 2016

AD, Alzheimer's disease; ALS, amyotrophic lateral sclerosis; Aβ, amyloid-beta; CMA, chaperone-mediated autophagy; GSDMD, gasdermin D; HD, Huntington's disease; ICH, intracerebral hemorrhage; IS, ischemic stroke; MCAO/R, middle cerebral artery occlusion/reperfusion; MLKL, mixed lineage kinase domain-like protein; OM-MSCs, olfactory mucosa-derived mesenchymal stem cells; PD, Parkinson's disease; RIPK, receptor-interacting protein kinase; SAH, subarachnoid hemorrhage; SCI, spinal cord injury.

## 5 Therapeutic approaches

### 5.1 Targeting individual PANoptosis-related pathways

Current therapeutic research in neurological diseases predominantly focuses on targeting specific branches within the PANoptosis network, such as inflammasome activation, caspase-1-dependent maturation of inflammatory cytokines, or RIPK1-RIPK3-MLKL-mediated necroptosis. This approach has practical value because these pathways have relatively clear inhibitors and measurable biomarkers. Still, its limitation should be noted. Blocking one pathway does not mean that PANoptosis as a whole has been fully inhibited. For this reason, single-pathway interventions should be described more cautiously. They represent pathway-level regulation rather than complete blockade of PANoptosis.

#### 5.1.1 Targeting NLRP3 inflammasome

NLRP3 has received considerable attention as a therapeutic target because it functions upstream in inflammatory cell death. Once activated, NLRP3 contributes to inflammasome assembly and caspase-1 activation. These events then lead to IL-1β and IL-18 maturation and GSDMD-mediated membrane pore formation. This makes it highly relevant to the pyroptotic branch of PANoptosis. MCC950, also known as CP-456,773, is a selective small-molecule inhibitor of the NLRP3 inflammasome. Its main feature is to block NLRP3 activation and inflammasome formation, rather than broadly suppressing all inflammasomes (Coll et al., 2015). A study showed that MCC950 directly acts on the Walker B motif within the NACHT domain of NLRP3 and inhibits adenosine triphosphate hydrolysis, thereby preventing activated NLRP3 from proceeding to inflammasome assembly (Coll et al., 2019). In studies of neurological diseases, the protective effects of MCC950 show a relatively consistent pattern. In chronic neurodegenerative diseases, it mainly reduces glial inflammation, oxidative stress and chronic neuronal loss, whereas in acute brain injury and SCI, it more prominently reduces edema, barrier disruption, inflammatory cytokine release and early neurological deficits. For example, in AD- and PD-related models, MCC950 attenuates neuronal injury induced by Aβ- or α-syn-related inflammatory stimulation through inhibition of NLRP3-caspase-1-GSDMD-related signaling, accompanied by improvements in learning and memory, motor behavior or DA neuronal survival (Gordon et al., 2018; Li et al., 2020). This effect is not limited to chronic degenerative disorders. In acute neurovascular injuries such as IS (Ismael et al., 2018), SAH (Dodd et al., 2021) and ICH (Ren et al., 2018), MCC950 can also attenuate the inflammatory cascade caused by excessive NLRP3 inflammasome activation, thereby reducing brain edema, infarct- or hemorrhage-related secondary injury and neurological dysfunction. Furthermore, in HD and SCI studies, the effects of MCC950 also point to the same central mechanism. By suppressing NLRP3 inflammasome activity, MCC950 reduces injury-related signals such as IL-1β, IL-18 and reactive oxygen species (ROS), while preserving more neuronal structures and synapse-related proteins, ultimately improving motor or histological outcomes (Jiao et al., 2020; Chen et al., 2022; Qin et al., 2025). These findings support NLRP3 as a useful upstream target, but they do not prove that the whole PANoptosis network is shut down. In future studies, NLRP3 inhibition should be combined with detection of GSDMD-N, cleaved caspase-3, caspase-8, RIPK1, RIPK3, and phosphorylated MLKL. This would help determine whether NLRP3 inhibition only reduces pyroptosis or also weakens broader PANoptosis-related pathway crosstalk.

#### 5.1.2 Inhibiting caspase-1-dependent pathways

Caspase-1 is an important effector activated by inflammasomes. Its main function is to promote the maturation and release of inflammatory cytokines. Caspase-1 also cleaves GSDMD, which causes membrane pore formation. Through this process, injured cells can develop an inflammatory cell death phenotype (Tweedell et al., 2024). Therefore, the significance of caspase-1 inhibition is not simply to reduce IL-1β, but to decrease the overall activity of the NLRP3-caspase-1-GSDMD inflammatory cell death branch. Belnacasan (VX-765) is a commonly used caspase-1 inhibitor in neurological disease research. Its protective effect may connect cognitive impairment, synaptic injury and neuroinflammation. In J20 AD mice, VX-765 dose-dependently improved episodic memory, spatial memory and hyperactivity. Cognitive impairment reappeared after drug withdrawal, and behavioral improvement returned after re-administration, indicating that caspase-1 activity is not a transient bystander but may continuously participate in disease maintenance (Flores et al., 2018). Furthermore, in aged AD mice, VX-765 still improved cognition and was accompanied by recovery of dendritic spines and synaptophysin, while its effect on total Aβ burden was limited. This suggests that the main value of caspase-1 inhibition may lie more in synaptic protection and reduction of inflammatory cell death pressure (Flores et al., 2022). In acute injury models, caspase-1 inhibition also shows a similar effect of reducing inflammatory amplification. For example, VX-765 reduced infarct volume and neurological deficits in IS, and promoted the shift of microglia and macrophages toward an M2-like phenotype (Li et al., 2019). In SCI, repeated VX-765 administration reduced caspase-1, IL-1β and IL-18 levels, and improved neurological recovery, indicating that caspase-1 is an important link between post-injury cytokine release and motor dysfunction (Chen et al., 2021). However, caspase-1 inhibition mainly targets the inflammasome-pyroptosis branch. It may not fully block caspase-8-dependent apoptosis or RIPK3-MLKL-dependent necroptosis. For this reason, caspase-1 inhibitors should be considered as important tools for reducing inflammatory amplification.

#### 5.1.3 Blocking the RIPK1-RIPK3-MLKL necroptosis axis

The RIPK1-RIPK3-MLKL axis represents the necroptotic branch of the PANoptosis network. It is especially relevant to neurological diseases because necroptosis causes membrane damage and releases intracellular contents that further stimulate inflammation (Samir et al., 2020). Nec-1 is a classical RIPK1 inhibitor. In IS rats, it reduced p-RIPK1, RIPK3, MLKL, p-MLKL and mature IL-1β levels, and attenuated post-ischemic brain injury, indicating that RIPK1 inhibition not only affects necroptosis but may also indirectly reduce cytokine release (Deng et al., 2019). This protective effect is also observed in hemorrhagic injury. In ICH mice, Nec-1 reduced RIPK1-RIPK3 interaction, propidium iodide-positive cell death, microglial activation, TNF-α and IL-1β expression, and improved brain edema and neurological function (Su et al., 2015). In SAH rats, Nec-1 reduced brain swelling and lesion size by inhibiting RIPK3-mediated necroptosis and alleviating blood-brain barrier disruption, further suggesting that this axis may participate in the spread of secondary injury after hemorrhage (Chen et al., 2019). In chronic neurodegenerative diseases, the significance of RIPK1 inhibition is not limited to reducing cell death. For example, in APP/PS1 mice and human AD samples, RIPK1 participated in disease-associated microglial phenotypic transition by inducing Cst7, and affected lysosomal function and phagocytic capacity (Ofengeim et al., 2017). In PD models, increased RIPK1, RIPK3 and MLKL are associated with DA neuronal loss, while Nec-1s reduces RIPK1 activation, mitochondrial dysfunction, ROS generation and inflammatory responses in 1-methyl-4-phenylpyridinium (MPP^+^)-treated SH-SY5Y cells and MPTP-treated mice (Lin et al., 2020; Liu et al., 2021). Furthermore, RIPK1 and RIPK3 participate in axonal degeneration, inflammation and necroptosis. This evidence comes from optineurin-deficiency models, SOD1-G93A mice and human ALS pathological samples, making RIPK1 a representative target moving from mechanistic research toward translational research (Ito et al., 2016). SAR443060, also known as SAR443060 (DNL747), is a central nervous system-penetrant RIPK1 inhibitor. In phase I/Ib studies involving healthy participants, AD patients and ALS patients, it showed cerebrospinal fluid exposure, pharmacokinetic properties and target engagement (Vissers et al., 2022). However, these findings should still be interpreted as early feasibility evidence rather than clinical efficacy evidence.

### 5.2 PANoptosis-specific interventions

Compared with targeting a single branch, PANoptosis-specific intervention aims to affect the shared platform or terminal process that connects pyroptosis, apoptosis, and necroptosis. This idea is more consistent with the definition of PANoptosis, but the current evidence in neurological diseases is still limited. At present, the strongest evidence usually comes from studies that show coordinated activation of multiple death pathways together with protein interaction, colocalization, or rescue experiments.

#### 5.2.1 Interfering with PANoptosome assembly

If the NLRP3-caspase-1-GSDMD and RIPK1-RIPK3-MLKL axes represent important branches of the PANoptosis network, then direct interference with PANoptosome assembly is closer to the core of PANoptosis-targeted therapy (Pandeya and Kanneganti, 2024). Relatively direct evidence in neurological diseases currently comes from SAH research. This study found that PGAM5 shifted from mitochondria-related sites to the cytosol, bound RIPK1 and promoted RIPK1 Ser166 phosphorylation, thereby driving RIPK1-PANoptosome activation (Jiajia et al., 2025). In this study, reducing PGAM5 decreased neuronal PANoptosis markers and improved both short-term and long-term neurological outcomes in rats, making the PGAM5-RIPK1-PANoptosome axis one of the few relatively clear direct PANoptosis intervention targets in SAH (Jiajia et al., 2025). The value of this discovery lies in its proposal of a composite mechanism that accounts for the co-variation of these biomarkers, bringing us closer to the essence of PANoptosis than the isolated detection of NLRP3, cleaved caspase-3, or p-MLKL.

### 5.3 Indirect or upstream modulators of PANoptosis

Many treatments do not directly target the PANoptosome or a single death-execution molecule. Instead, they act on upstream conditions that make PANoptosis more likely to occur, such as microglial overactivation, oxidative stress, mitochondrial injury, lysosomal dysfunction, and persistent cytokine release. These approaches are less specific, but they may be useful in neurological diseases because neural injury usually develops through several overlapping mechanisms rather than one isolated pathway.

#### 5.3.1 Regulating microglial activation

Microglia are a key cell type in PANoptosis-related intervention in the nervous system, because they can release IL-1β, IL-18 and TNF-α, and can also influence neuronal fate through phagocytosis, antigen processing, lysosomal function and repair responses. In AD, microglia are not only a source of inflammation. They also determine whether Aβ clearance and the synaptic environment continue to deteriorate. For example, NLRP3-dependent microglial training impaired Aβ clearance and aggravated cognitive decline (He et al., 2020). RIPK1 can also affect microglial states through mechanisms beyond direct cell death. In APP/PS1 mice, RIPK1-induced Cst7 expression affected lysosomal pathways and phagocytic capacity, thereby promoting disease-associated microglial responses (Ofengeim et al., 2017). In acute injury, the meaning of microglial regulation is more focused on controlling secondary inflammatory spread. For example, in IS, VX-765 promoted the transition of microglia and macrophages toward an M2-like phenotype while suppressing NF-κB activation (Li et al., 2019). In PD, the RIPK1 inhibitors Nec-1 and Nec-1s reduced LPS-induced neuroinflammation and MPTP-induced microglial activation, suggesting that RIPK1 may regulate both cell death signaling and glial inflammatory responses (Kim et al., 2023).

#### 5.3.2 Regulating oxidative stress, mitochondrial injury and lysosomal function

Oxidative stress, mitochondrial injury and lysosomal dysfunction are often located upstream of PANoptosis-related pathways, because they can generate danger signals, release mitochondria-related damage molecules, and weaken the ability of cells to clear damaged proteins and organelles. In acute neural injury, these upstream changes can amplify localized cellular stress into inflammatory cell death. For example, after SCI, lysosomal damage caused accumulation of RIPK1 and RIPK3 proteins and increased neuronal sensitivity to necroptosis (Liu et al., 2018). In PD-related experiments, Nec-1s reduced RIPK1 activation and also decreased ROS generation and mitochondrial dysfunction, suggesting that necroptosis inhibition and mitochondrial protection may reinforce each other (Liu et al., 2021). In SAH, salvianolic acid B (SalB) reduced oxidative injury through Nrf2- and sirtuin 1 (SIRT1)-related pathways, as shown by decreased ROS and lipid peroxidation, and restored glutathione peroxidase, glutathione and superoxide dismutase activities (Zhang et al., 2018). The advantage of these upstream regulatory strategies is their broad action range. They may reduce inflammasome activation and also weaken persistent activation of the RIPK1-RIPK3-MLKL axis.

#### 5.3.3 Natural products and multi-target compounds

Natural products and multi-target compounds have attracted attention in neurological diseases because they often do not act on a single protein only. Instead, they may simultaneously affect inflammasomes, oxidative stress, mitochondrial function and glial responses. Echinacoside is a natural phenylethanoid glycoside. In an MPTP-induced PD model, it inhibited microglial NLRP3, caspase-1 and IL-1β signaling, and attenuated DA neuronal injury, indicating that natural products can also influence neuronal fate through the inflammasome entry point (Gao et al., 2020). Parthenolide is a natural compound related to NF-κB and NLRP3 regulation. In a 3-nitropropionic acid-induced HD-like rat model, it improved motor, cognitive and anxiety-like behaviors, and reduced NLRP3, NF-κB and microglial activation (Noureldeen et al., 2024). Furthermore, SalB mainly shows antioxidant and neuroprotective effects in SAH models. By regulating Nrf2- and SIRT1-related pathways, it reduces ROS, lipid peroxidation and oxidative stress injury, providing an example of modifying the upstream environment of PANoptosis (Zhang et al., 2018). Compared with relatively clear modulators of PANoptosis-related pathways such as MCC950, VX-765 or Nec-1, the advantage of natural products lies in multi-step regulation, while their limitation is that the targets are less specific and mechanistic attribution can be more easily confounded. Therefore, future research on natural products must simultaneously detect indicators such as GSDMD-N, cleaved caspase-3, caspase-8, p-RIPK1, p-RIPK3, p-MLKL, and lactate dehydrogenase release to support conclusions regarding PANoptosis intervention, rather than relying solely on the detection of inflammatory factors or oxidative stress markers.

In addition, because PANoptosis itself is characterized by pathway crosstalk, future treatment may not be suitable for relying on a single-point inhibitor alone. Instead, combination intervention should be selected according to disease type, injury stage and major pathological drivers. The rationale for combination therapy has already appeared in some experiments. For example, in ICH research, the NLRP3 inhibitor Dapansutrile (OLT1177) reduced brain edema, blood-brain barrier disruption, apoptosis and downstream inflammasome protein elevation, while combination with the caspase-1 inhibitor VX-765 may further strengthen control of inflammasome-related injury (Fang et al., 2024; Zhou et al., 2025). In SCI, OLT1177 also reduced IL-1β- and IL-18-related inflammatory responses by inhibiting the NLRP3 inflammasome, and preserved myelin and motor function, indicating that the same intervention node may have shared value across different acute injuries (Amo-Aparicio et al., 2022).

**Figure 3:**
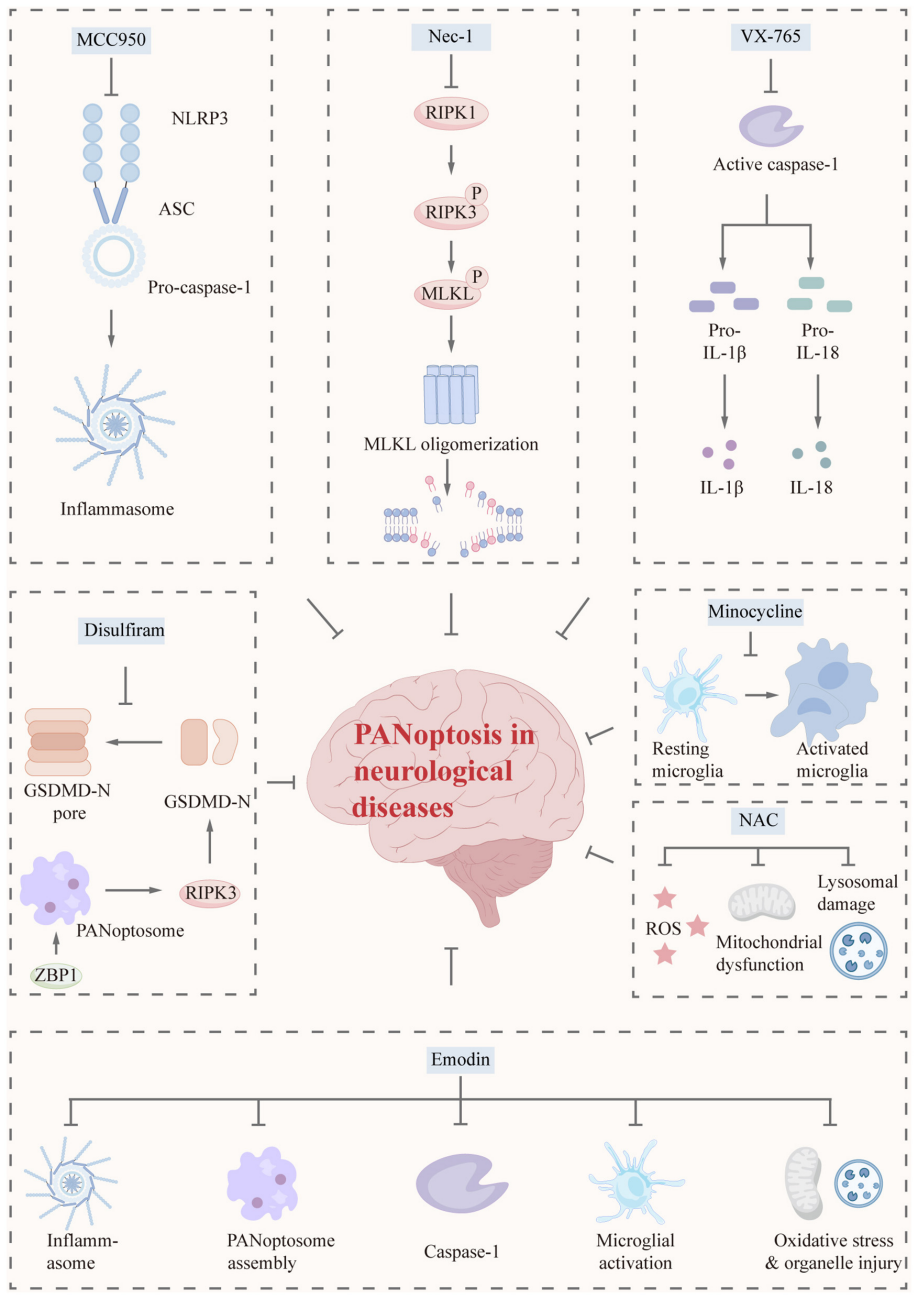
Therapeutic targeting of PANoptosis-associated inflammatory injury in neurological diseases. Neurological diseases are frequently accompanied by convergent activation of inflammasome signaling, necroptotic membrane disruption, PANoptosome assembly, pyroptotic pore formation, microglial activation, oxidative stress, and organelle damage, all of which may amplify neuroinflammation and neuronal injury. The NLRP3 inflammasome pathway is initiated by NLRP3 activation, recruitment of apoptosis-associated speck-like protein containing a caspase recruitment domain (ASC), and pro-caspase-1 assembly, leading to inflammasome formation and downstream inflammatory responses. MCC950 suppresses this process by inhibiting NLRP3 inflammasome activation. Necroptosis is primarily mediated by receptor-interacting serine/threonine-protein kinase 1 (RIPK1), receptor-interacting serine/threonine-protein kinase 3 (RIPK3), and mixed lineage kinase domain-like protein (MLKL). Following RIPK1–RIPK3 signaling, phosphorylated MLKL oligomerizes and disrupts plasma membrane integrity, thereby promoting inflammatory cell death; Nec-1 interferes with this cascade by inhibiting RIPK1-dependent necroptotic signaling. Caspase-1 activation promotes the maturation of pro-interleukin-1β (pro-IL-1β) and pro-interleukin-18 (pro-IL-18) into IL-1β and IL-18, respectively, thereby amplifying neuroinflammation; VX-765 attenuates this process by inhibiting caspase-1 activity. Gasdermin D N-terminal fragment (GSDMD-N)-mediated pore formation represents a critical execution step of pyroptosis and may also participate in PANoptosome-associated inflammatory cell death. Disulfiram inhibits GSDMD-N pore formation, thereby limiting pyroptotic membrane permeabilization and inflammatory mediator release. In parallel, Z-DNA-binding protein 1 (ZBP1), RIPK3, and PANoptosome assembly may coordinate the activation of pyroptotic, apoptotic, and necroptotic molecular programs under pathological conditions. Microglial activation further sustains neuroinflammation, whereas minocycline suppresses the transition of resting microglia toward an activated pro-inflammatory state. N-acetylcysteine (NAC) reduces reactive oxygen species (ROS) accumulation and alleviates mitochondrial dysfunction and lysosomal damage, thereby mitigating oxidative stress- and organelle injury-associated neuronal damage. Emodin exerts multi-target regulatory effects by suppressing inflammasome activation, PANoptosome assembly, caspase-1 activation, microglial activation, oxidative stress, and organelle injury. Collectively, these interventions highlight multiple pharmacological entry points for modulating PANoptosis-associated inflammatory cell death, oxidative injury, and neuroinflammatory amplification in neurological diseases. ASC, apoptosis-associated speck-like protein containing a caspase recruitment domain; GSDMD-N, N-terminal fragment of gasdermin D; IL-1β, interleukin-1β; IL-18, interleukin-18; MCC950, NLRP3 inflammasome inhibitor; MLKL, mixed lineage kinase domain-like protein; NAC, N-acetylcysteine; Nec-1, necrostatin-1; NLRP3, NOD-like receptor family pyrin domain-containing 3; PANoptosome, PANoptosis-associated multiprotein complex; RIPK1, receptor-interacting serine/threonine-protein kinase 1; RIPK3, receptor-interacting serine/threonine-protein kinase 3; ROS, reactive oxygen species; VX-765, caspase-1 inhibitor; ZBP1, Z-DNA-binding protein 1.

Even so, multi-target or combined intervention should be evaluated cautiously. Excessive inhibition of inflammatory cell death may reduce harmful inflammation, but it may also interfere with pathogen defense, damaged-cell clearance, and tissue repair. Future studies should therefore measure not only short-term molecular markers such as GSDMD-N, cleaved caspase-3, p-RIPK1, p-RIPK3, and p-MLKL, but also long-term behavior, tissue repair quality, and safety (Figure 3).

## 6 Conclusions

In conclusion, PANoptosis provides a more integrated perspective for understanding cell death and inflammatory amplification in neurological diseases. PANoptosis differs from the separate analysis of pyroptosis, apoptosis, or necroptosis. It focuses on how these death pathways activate one another and amplify injury within the same pathological environment. In chronic neurodegenerative diseases, including AD, PD, HD, and ALS, several long-term stressors may contribute to this process. These include abnormal protein aggregation, sustained glial activation, mitochondrial damage, and synaptic dysfunction. Together, they can maintain inflammatory cell death over time. A different pattern may occur in acute neurological injuries, such as IS, SAH, ICH, and SCI. In these conditions, ischemia, hypoxia, blood degradation products, mechanical trauma, oxidative stress, and barrier disruption can rapidly trigger more than one death pathway. For this reason, neuronal loss should not be explained by a single mode of cell death alone. It is more likely to result from the combined effects of inflammation, metabolic imbalance, organelle injury, and multiple regulated cell death pathways.

Mechanistically, the main value of PANoptosis is that it explains how multiple death signals are integrated through shared molecular nodes. Molecules such as NLRP3, caspase-1, caspase-8, RIPK1, RIPK3, MLKL, GSDMD, and NINJ1 do not act in isolation. Instead, they may form dynamic connections across different diseases, cell types, and disease stages. The PANoptosome can be understood as a signal-converging platform that links danger-signal recognition, inflammasome activation, mitochondrial injury, pore formation, and terminal membrane rupture. Thus, PANoptosis is better regarded as a flexible inflammatory cell death network rather than a fixed and linear molecular pathway. Therapeutically, PANoptosis suggests that future neuroprotective strategies should not focus on a single target alone. Inhibition of the NLRP3 inflammasome, blockade of the RIPK1/RIPK3/MLKL axis, regulation of caspase-8 activity, improvement of mitochondrial and lysosomal function, reduction of oxidative stress, and control of excessive microglial activation may all reduce PANoptosis-related injury to different degrees. However, complete blockade of cell death may also interfere with the clearance of damaged cells and tissue repair. Therefore, a more reasonable strategy is not simply to “shut down” PANoptosis, but to regulate its intensity within an appropriate disease stage and therapeutic window, thereby reducing excessive inflammatory spread while preserving necessary defense and repair functions.

Overall, PANoptosis provides a new framework for neurological disease research, shifting the focus from a single death pathway to a broader network of cell death and inflammation. It helps explain why neuronal death, glial inflammation, blood-brain barrier or blood-spinal cord barrier disruption, and functional deterioration often occur together in many neurological diseases.

## 7 Limitations and future perspectives

Although PANoptosis is a useful framework for connecting pyroptosis, apoptosis and necroptosis in neurological diseases, the field still lacks a unified marker system that separates true PANoptosis from the loose coexistence of several death pathways. Many studies still judge PANoptosis by measuring NLRP3, cleaved caspase-1, cleaved caspase-3, RIPK3, p-MLKL and GSDMD-N separately, whereas Christgen et al. showed that stronger evidence requires combined pathway activation together with protein interaction supporting PANoptosome formation (Christgen et al., 2020). This limitation is important in the nervous system, because neurons, microglia, astrocytes, endothelial cells and infiltrating immune cells may show different death patterns under the same injury condition. Future studies should use a minimum evidence set that includes co-activation of the three death branches in the same lesion, interaction or colocalization among core components, and rescue by inhibiting key nodes.

Another limitation is that direct evidence for PANoptosome assembly remains weak in most neurological diseases. Infection studies showed that AIM2 can form a complex with pyrin, ZBP1, ASC, caspase-1, caspase-8, RIPK1, RIPK3 and FADD, and heme-related studies showed that NLRP12 can organize PANoptosome activation and tissue pathology (Lee et al., 2021; Sundaram et al., 2023). These studies provide a useful template, but similar co-immunoprecipitation, proximity ligation, expansion microscopy or spatial proteomics evidence is still uncommon in AD, PD, IS, ICH, HD, SCI and ALS. Current neurological evidence often stays at the marker level, such as NLRP3 activation in APP/PS1 mice and human AD brain samples, or α-syn-induced inflammasome activation in microglia and PD models treated with MCC950 (Heneka et al., 2013; Gordon et al., 2018). Necroptosis-related studies have also shown disease relevance, including RIPK1-driven microglial dysfunction in AD, RIP1/RIP3/MLKL-mediated dopaminergic neuronal necroptosis in MPTP-treated mice, and RIPK1-mediated axonal degeneration in ALS models (Ofengeim et al., 2017; Lin et al., 2020). However, these results mainly prove that single branches of the PANoptosis network are active, and they do not prove that a PANoptosome is assembled in the same cell.

A third challenge is the gap between animal models and human disease. Mouse models of ischemia, SAH, toxin-induced PD or genetic ALS allow timed sampling and genetic intervention, but they only partly reproduce the long disease course, mixed pathology, aging background and treatment history seen in patients. The recent SAH study on PGAM5 provides useful mechanistic evidence. In rats, PGAM5-dependent RIPK1 phosphorylation was linked to RIPK1-PANoptosome activity and better neurological outcomes. However, it remains unclear whether the same axis can be detected in human SAH tissue or cerebrospinal fluid (Jiajia et al., 2025). Human studies have started to address this issue. In patients with aneurysmal SAH, NLRP3 overactivation and higher levels of IL-1β, IL-18, GSDMD, and tissue factor were reported in monocytes and plasma. In PD patients, systemic NLRP3 activation was also associated with disease severity (Fan et al., 2020; Díaz-García et al., 2023).

Disease stage is another key issue. Acute injuries such as IS and SAH have an early inflammatory phase and a later repair phase, whereas AD, PD, HD and ALS progress over years with changing glial states and neuronal vulnerability. For this reason, inhibiting PANoptosis may be protective during excessive inflammatory amplification, but broad or late inhibition may disturb debris clearance, host defense or tissue repair. Early clinical studies of the central nervous system-penetrant RIPK1 inhibitor SAR443060 showed cerebrospinal fluid exposure and target engagement in AD and ALS participants, but they also remind us that target engagement is only the first step toward proving long-term benefit and safety (Vissers et al., 2022). Furthermore, the field needs stronger cell type-specific evidence. Single-nucleus RNA sequencing of human midbrain has shown cell type-resolved changes across glial and neuronal populations in PD, suggesting that bulk tissue measurements can hide the exact cells carrying PANoptosis-related signals (Smajić et al., 2022). Future work should integrate single-cell and spatial methods with lineage tracing, conditional knockout models and region-specific sampling, so that PANoptosis-targeted therapy can become stage-matched and cell type-targeted.
